# Optimization of the Quality of Reclaimed Water from Urban Wastewater Treatment in Arid Region: A Zero Liquid Discharge Pilot Study Using Membrane and Thermal Technologies

**DOI:** 10.3390/membranes15070199

**Published:** 2025-07-01

**Authors:** Maria Avramidi, Constantinos Loizou, Maria Kyriazi, Dimitris Malamis, Katerina Kalli, Angelos Hadjicharalambous, Constantina Kollia

**Affiliations:** 1 School of Chemical Engineering, National Technical University of Athens, Zografou Campus, 15780 Athens, Greece; conloizou@hotmail.com (C.L.); kyriazimaria@mail.ntua.gr (M.K.); dmalamis@chemeng.ntua.gr (D.M.); dinak@chemeng.ntua.gr (C.K.); 2 Direct Local Government Organization of Larnaca, 12 Adamantiou Korai Str., Larnaca 6302, Cyprus; kkalli@eoal.org.cy (K.K.); ahadjicharalambous@eoal.org.cy (A.H.)

**Keywords:** water reuse, zero liquid discharge, wastewater treatment, water recovery, membrane technologies, thermal technologies

## Abstract

With water availability being one of the world’s major challenges, this study aims to propose a Zero Liquid Discharge (ZLD) system for treating saline effluents from an urban wastewater treatment plant (UWWTP), thereby supplementing into the existing water cycle. The system, which employs membrane (nanofiltration and reverse osmosis) and thermal technologies (multi-effect distillation evaporator and vacuum crystallizer), has been installed and operated in Cyprus at Larnaca’s WWTP, for the desalination of the tertiary treated water, producing high-quality reclaimed water. The nanofiltration (NF) unit at the plant operated with an inflow concentration ranging from 2500 to 3000 ppm. The performance of the installed NF90-4040 membranes was evaluated based on permeability and flux. Among two NF operation series, the second—operating at 75–85% recovery and 2500 mg/L TDS—showed improved membrane performance, with stable permeability (7.32 × 10^−10^ to 7.77 × 10^−10^ m·s^−1^·Pa^−1^) and flux (6.34 × 10^−4^ to 6.67 × 10^−4^ m/s). The optimal NF operating rate was 75% recovery, which achieved high divalent ion rejection (more than 99.5%). The reverse osmosis (RO) unit operated in a two-pass configuration, achieving water recoveries of 90–94% in the first pass and 76–84% in the second. This setup resulted in high rejection rates of approximately 99.99% for all major ions (Cl^−^, Na^+^, Ca^2+^, and Mg^2+^), reducing the permeate total dissolved solids (TDS) to below 35 mg/L. The installed multi-effect distillation (MED) unit operated under vacuum and under various inflow and steady-state conditions, achieving over 60% water recovery and producing high-quality distillate water (TDS < 12 mg/L). The vacuum crystallizer (VC) further concentrated the MED concentrate stream (MEDC) and the NF concentrate stream (NFC) flows, resulting in distilled water and recovered salts. The MEDC process produced salts with a purity of up to 81% NaCl., while the NFC stream produced mixed salts containing approximately 46% calcium salts (mainly as sulfates and chlorides), 13% magnesium salts (mainly as sulfates and chlorides), and 38% sodium salts. Overall, the ZLD system consumed 12 kWh/m^3^, with thermal units accounting for around 86% of this usage. The RO unit proved to be the most energy-efficient component, contributing 71% of the total water recovery.

## 1. Introduction

Based on the United Nations (UN) World Water Development Report (WWDR) published in 2024, freshwater use has been growing by just under 1% per year [1]. The 70% of freshwater withdrawals globally are attributed to agriculture [2] with the demand for water in agriculture projected to increase by about 60% until 2050 [3]. Water preservation is a key priority to ensure sustainable management of water resources in both global and European contexts with the recycle and reuse of water being one of the main ways to address scarcity with the market size expected to grow at a compound annual growth rate (CAGR) of over 10% between 2024 and 2032 [4].

Reclaimed water (RW) along with the desalination and the water transfer are the main efficient alternative water resources [5]. Water reclamation (treatment or processing of wastewater to make it reusable with definable treatment reliability and meeting appropriate water quality criteria) and water reuse (the use of treated wastewater for a beneficial use, such as agricultural irrigation and industrial cooling) play a crucial role in addressing global water scarcity [6]. In this respect, the use of RW can mitigate water scarcity through its application in landscaping and municipal uses, industrial processes, aquifer recharge, and agricultural irrigation [7]. The 52% share of use of reclaimed water is mainly used for irrigation (agricultural and landscape) [8]. In regions where access to water is difficult, the reuse of treated wastewater is preferred because it is more feasible than other alternatives [9,10].

Water reuse is widely practiced in Mediterranean countries like Spain, France, Greece, Cyprus, and Malta, where agricultural and drinking water shortages have been a persistent challenge for many years [11]. Cyprus faces severe water scarcity due to frequent droughts, limited water resources, and increasing demand from population growth, tourism, and lifestyle changes. With a Water Exploitation Plus Index of approximately 71.4% for 2024 [12], water supply is prioritized for drinking purposes, while the agricultural sector—being the next larger consumer—often experiences irrigation cuts of up to 70% during drought periods, based on information from the Water Development Department. Climate change is expected to further worsen water availability, necessitating the adoption of alternative water sources such as desalinated water for drinking water and reclaimed water for irrigation and other uses. Currently, recycled water accounts for 11% of irrigation needs [13], with projections to increase to 25% within the next seven years through new infrastructure projects. The government promotes tertiary wastewater treatment through the Code of Agricultural Practice [14], ensuring high-quality reclaimed water for irrigation, groundwater recharge, and municipal green spaces.

The EU Directive 91/271/EEC and Regulation (EU) 2020/741 are the two key policy frameworks governing the safe and sustainable use of reclaimed water in agriculture after secondary treatment and disinfection (tertiary treatment) is implemented to meet the required quality standards. Except for the disinfection, there are different treatments that can be utilized to meet the required standards. As studied by Chao Chen [15], various treatment technologies have been explored, each with distinct mechanisms, efficiencies, and limitations. Membrane-based filtration methods, such as microfiltration (MF), ultrafiltration (UF), and nanofiltration (NF), effectively remove microorganisms. MBR (membrane bioreactors) integrate biological treatment with filtration, enhancing virus removal [16].

The impact of high soil salinity is a major concern when RW is used for irrigation [17]. The excessive and unregulated application of irrigation water with high electrical conductivity (greater than 2.9 mS/cm) and a high sodium adsorption ratio (SAR) can lead to the accumulation of ions in plant roots and leaves, potentially causing toxicity, crop damage, and reduced yields [18,19]. The European Commission’s guidelines for implementing Regulation 2020/741 on minimum requirements for water reuse highlight salinity—measured through total dissolved solids and electrical conductivity—as a priority factor due to its potential negative impact when RW is used for agricultural irrigation. The effects of salinity in irrigation with RW were studied by Oussama Mounzer et.al [20], revealing that the salinity load in the irrigation water significantly reduced the available fraction of soil water. This, in turn, exposed the root system to increased osmotic stress, which determines the plant water uptake in the field, and intensified the risk of ion toxicity. Leogrande R. et al. [21] studied the impact of irrigation with high salinity RW (within 10 years of irrigation) and found that higher values of conductivity (EC) and Na content were observed in the soil receiving reclaimed water (5.4 dS m^−1^ and 358 mg kg^−1^, respectively) in comparison to fresh water (FR) (3.6 dS m^−1^ and 66 mg kg^−1^, respectively).

The advanced treatment of reclaimed water in water stress countries could act as a mitigation measurement with suitable process integration. In the literature, different membrane technologies have been evaluated for their feasibility to be integrated for water reclamation processes from wastewater treatment plants. Gideon Oron et al. [22] evaluated the integration of ultrafiltration (UF) and reverse osmosis (RO) technologies for treating secondary treated water derived from wastewater plants with 8 m^3^/h pilot system capacity. The reclaimed water from the RO met the limits of irrigation requirements, and the different tested configurations estimated the best conditions for crop corn production. Similarly, Mohamed F. Hamoda et al. [23] evaluated the performance of UF and RO systems, with a design capacity of 425,000 m^3^/d, for secondary treated wastewater, and the analysis showcased that the reclaimed water met the water quality standards and that the system configuration is reliable. The membrane technologies that can be utilized are considered advanced processes since they are installed not for the operation of the wastewater treatment plant but to shift wastewater to high-quality reclaimed water [24]. The previously mentioned studies performed as tertiary treatment on the wastewater hybrid NF/RO technologies without considering the untreated effluents that are generated by the process.

Although the feasibility of treating high salinity water with different Zero Liquid Discharge (ZLD) technologies has been extensively reviewed [25], and the feasibility of installing different systems has been extensively discussed [26,27], few studies have investigated the operational feasibility of ZLD systems in wastewater treatment plants, which aim to recover water without losses, minimize untreated discharges, and ensure a sustainable wastewater treatment approach. ZLD systems are considered a very promising solution for treating saline effluents, but the challenges they present during operation need to be considered. The integrated membrane units, due to their high concentrations of Ca^2+^, Mg^2+^, and CO_3_^−^, need to be operated with caution to avoid fouling and scaling, which would otherwise lead to high operating expenses (OPEX) from membrane replacement [28]. For thermal units, high energy consumption is one of the main parameters influencing the OPEX of a ZLD system, usually accounting for over 80% of the total OPEX alongside high CAPEX [29]. Addressing these challenges requires pilot-scale installations, as these provide critical technical insights and operational data to support informed decision-making for full-scale deployment.

Focusing on Cyprus, an arid region, a ZLD system was integrated and operated at Larnaca Wastewater Treatment Plant (WWTP), treating reclaimed water from the tertiary treatment stage (after MBR treatment and before chlorination). The treatment of the reclaimed water was implemented with the utilization of membrane and thermal technologies. The study aims to assess the effectiveness and technical viability of the ZLD system in treating high-salinity wastewater, recovering valuable resources (water and salts) that could be utilized within the plant.

## 2. Materials and Methods

### 2.1. Inflow Characteristics

The analysis of MBR effluent from the WWTP of Larnaca was crucial for the design of all pilot systems. As shown in Table 1, the effluent exhibited an electrical conductivity (EC) of approximately 4.2 mS/cm, which can be mainly attributed to the presence of Na^+^ and Cl^−^ ions, with an overall sodium chloride content around 1.5 g/L. Irrigation with reclaimed water usually accelerates the deterioration of soil properties, as the water has a higher concentration of Na^+^ than Ca^2+^ and Mg^2+^, resulting in an increased sodium adsorption ratio (SAR), which can lead to sodicity hazards [30]. High sodicity (high SAR) can affect soil structure and quality, thereby reducing crop yield. Along with high electrical conductivity (EC), it is one of the two main factors that can impact crop production [31]. According to the US EPA, EC is the most important factor for the quality of reclaimed water for different agricultural and/or industrial uses, as it is the main indicator of salinity levels in water and can influence the soil’s osmotic potential and specific ion toxicity [32].

Furthermore, the effluent had a COD concentration of 20 mg/L. This parameter was carefully considered during the design of the ZLD system, since high levels of organics can cause operational issues in membrane-based processes. To address this, the NF unit was employed. This reduced the COD content. It also separated monovalent from divalent ions, protecting the downstream RO membranes.

Additionally, the presence of total suspended solids (TSS) was recognized as a potential challenge for the NF membranes. To mitigate this issue, the system incorporated pre-filtration units comprising cartridge filters to reduce the particulate load prior to entering the NF unit. These were supported by periodic backwashing and chemical cleaning protocols to prevent membrane fouling and maintain optimal performance.

### 2.2. Description of the ZLD Pilot Design

**Figure 1:**
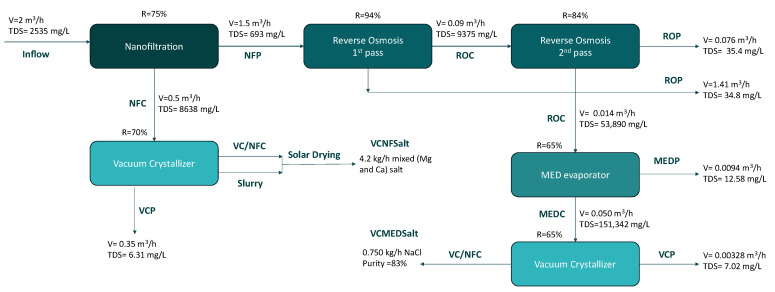
Process and mass balance of the installed unit.

The pilot-scale implementation of the proposed process was designed to treat a daily inflow of 36 to 48 m^3^ (equivalent to 1.5–2 m^3^/h) of water from the tertiary treatment stage of the Larnaca Wastewater Treatment Plant (WWTP) (Figure 1). The integrated pilot system treatment began with a nanofiltration (NF) unit, which served as a pre-treatment step to remove divalent ions (Mg^2+^, Ca^2+^, SO_4_^2−^) and protect downstream membrane processes from potential scaling and fouling. The NF unit generated two streams: the NF concentrate (NFC), rich in divalent ions, and the NF permeate (NFP), containing monovalent ions. The NFP stream was processed through a reverse osmosis (RO) unit, which removed the remaining monovalent ions, producing RO permeate (ROP) as purified water and RO concentrate (ROC) as a highly saline waste stream. The ROC was then directed to the evaporation step, which employed a multi-effect distillation (MED) unit to further concentrate the brine. This process produced two outputs: distillate water MED permeate (MEDP) and a high-concentration brine, the MED concentrate (MEDC). As a final step, a vacuum crystallization (VC) unit was installed and during the pilot operation treated two different streams: the MEDC and the NFC streams, aiming to recover high purity NaCl salt (VCMEDSalt) and mixed salts VCNFSalt, respectively, with the system producing distilled water (VCP) from both treatments.

### 2.3. Description of the ZLD Pilot Design

#### 2.3.1. Membrane Technologies/Equipment and System Description

The pre-treatment stage is crucial in a ZLD system, significantly impacting the overall recovery and operational costs. Approximately 50% of pre-treatment technologies involve membrane processes like microfiltration (MF), ultrafiltration (UF), and nanofiltration (NF) [33,34].

The RO unit plays a critical role in a ZLD system by effectively removing monovalent ions and further concentrating the feedwater, thus preparing the high-salinity stream for subsequent evaporation and crystallization processes. As a high-pressure membrane process, RO can achieve salt rejection rates of up to 99% [35], making it one of the most efficient desalination technologies. However, its performance is highly dependent on proper pre-treatment, as fouling, scaling, and biofouling can significantly reduce membrane lifespan and increase operational costs [36].

**Figure 2:**
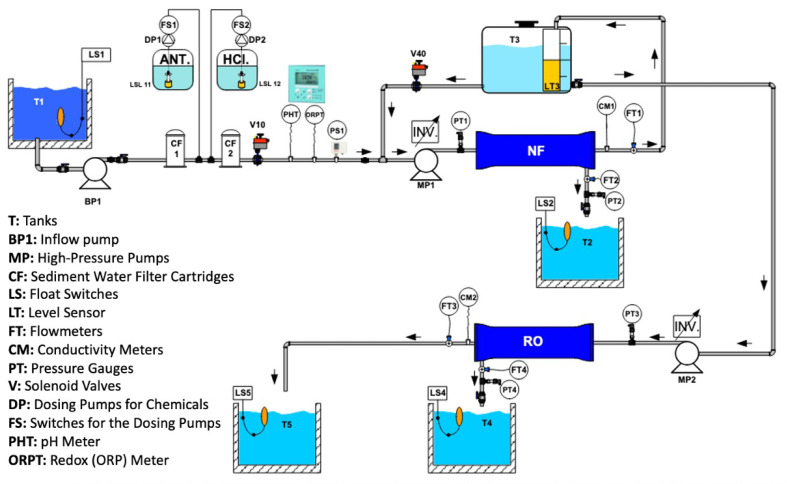
Schematic diagram of the installed RO and NF units.

At the installed pilot unit, as a pre-treatment, an NF unit was constructed and operated, and an RO unit was utilized for water recovery (Figure 2). The membrane systems were controlled through a programmable logic controller (PLC) interface.

##### Nanofiltration Unit

At the pilot operation, part of the outflow from the MBR of the WWTP was stored in a buffer tank and, through a feed pump, transferred to the NF unit. The water first passed through a pre-treatment stage consisting of 5-micron nominal cartridge filters. The feed pump provided initial pressurization, while an integrated high-pressure pump provided the necessary pressure for membrane filtration. The system included six NF90-4040 membranes (Table 2) connected in series, operating at a total treatment capacity of 2 m^3^/h. The system operated at different volumetric recovery rates to determine the optimal parameters. During operation, the membrane flux (f) fluctuated up to 6.67 × 10^−4^ m/s, while the average pressure drop (ΔP) was 87 kPa (kilopascal), remaining within the maximum permissible pressure drop of 90 kPa. To protect the membranes and maximize their lifespan, instead of the addition of antiscalants, the pH of the inflow was decreased from 8 to 6 with hydrochloric acid addition (HCl/30%). The membrane system was controlled through a programmable logic controller (PLC) interface, which connected numerous pressure, flow, water level, conductivity, pH, and energy transmitters at various locations within the system to receive essential data for process control. These sensors provided real-time process data, enabling continuous monitoring and control. The data collected from the PLC were used to track inlet and outlet pressure variations within the pressure vessel and to analyze the system’s energy requirements.

##### Reverse Osmosis Unit

The installed RO system operated using the NFP stream, which primarily contained monovalent ions. A high-pressure pump transferred the NFP flow from the NFP tank to the RO pressure vessel, operating at a maximum pressure of 4000 kPa (40 bar). The system was equipped with four FILMTEC LC HR 4040 membranes (Table 2), 4″ polyamide thin-film composite membranes, specifically designed for brackish water treatment. During pilot plant operation, the system maintained a permeate flux between 5.95 × 10^−4^ and 8.50 × 10^−4^, with an ΔP of 2000 kPa (20 bar). The LC-HR membranes were selected to be integrated into the pilot after investigation through the Water Application Value Engine (WAVE) and based on its designed salt reduction efficiency of 99.7%. Aiming to maximize the recovered water amount, a double pass operation was performed. The RO at its first pass was operated with high system recoveries ranging from 90% to 95% and for its second pass operated with recoveries ranging from 75% to 80%. Chemical cleaning was performed when the membrane pressure difference reached the upper limit specified in the membrane properties (0.78 bar), using diluted HCl (30%) and NaOH (50%). The key parameters such as pressure, flow rate, conductivity, and temperature were continuously monitored via the PLC interface. With achieved concentration more than 10 times greater with the operation of RO, the concentrated stream was treated by the following thermal technologies.

#### 2.3.2. Thermal Technologies/Equipment and System Description

The final process of a ZLD system is the crystallization process, in which the recovery of crystallized salts and distillate water takes place with the further treatment of the concentrated stream from the previous step. The crystallization can be achieved through different methods, depending on the type of crystallizer used. Several commercially available crystallizers operate based on batch or continuous processes [39] and employ cooling, evaporative, or vacuum-based principles [40]. The selection of the most suitable crystallizer depends on factors such as the desired end product and the characteristics of the feed.

Various evaporation units, including multi-effect distillation (MED) [41], multi-effect distillation with thermal vapor compression (MED-TVC) [42], multi-stage flash (MSF) [43], and mechanical vapor compression (MVC), are available in the market and play a crucial role in concentrating brine [44]. While thermal units have high energy demands due to phase changes, these demands can be minimized through proper design, operation, and maintenance [45]. Additionally, integrating these units with waste heat recovery systems can significantly reduce energy costs.

##### Multi Effect Distillation (MED) Evaporator

The installed MED evaporator unit is a multi-stage thermal desalination unit designed for efficient brine concentration and water recovery. Operating under vacuum, the system reduces the boiling point of water, aiming at energy efficiency. The system consists of a boiler for heating, inflow pumps, recirculation pumps for maintaining brine flow, and a vacuum pump to create the necessary pressure conditions. The evaporator unit operates under vacuum, with the first stage maintaining a pressure of approximately 40 kPa(a) and the second stage operating at a reduced pressure of 20 kPa(a) to facilitate efficient evaporation and heat recovery. A vacuum pump is used to maintain constant pressure. The pressure is regulated in each stage using the SCADA control system and electric valves. The brine is introduced into the system, heated through two effects, and concentrated, while excess heat can be recovered to optimize energy consumption. The heat to the first MED-stage is provided by hot water (80 °C) produced within the electric boiler and is recirculated via an in-line pump. Hot water (service) flows inside the heat exchanger tubes, transferring heat to the brine solution, which flows on the external surface of the tube bundle. The steam produced during the first stage is recovered and condensed in the second process. The service steam produced from the first effect flows inside the tubes of the second stage heat exchanger and condenses, transferring heat to the brine flowing on the external surface of the tubes. This allows water to evaporate from the brine during the second stage. The steam is then recovered in a water-cooled condenser in order to condensate this vapor. Following condensation of steam, heat contained in the stream is further recovered as it pre-heats the incoming brine, flowing through a plate heat exchanger.

The unit includes a cooling water circuit to regulate temperatures and prevent overheating, with adjustable flow rates based on cooling water temperature: 1 m^3^/h for water temperatures between 25–27 °C and 360 L/h for temperatures between 15–20 °C. The system operates effectively when the temperature of the heating source reaches the range of 70–80 °C, ensuring optimal thermal conditions for evaporation.

##### Vacuum Crystallizer (VC)

Regarding the final stage of the ZLD system, the VC unit is a concentrator that achieves the distillation of liquids at low temperatures through the combined effect of vacuum technology and the heat pump. The system was purchased by the Veolia company and is the model R 150, which operates as follows.

The concentrate effluent of the MED evaporator was fed in the VC unit through the pneumatic valve, which is controlled by a level control sensor located in the boiling chamber of the unit. Through a refrigerant circuit, the heat pump performs the expansion and compression of the Freon gas, providing both the calories needed to vaporize the liquid and the refrigerants needed to condense it. The heating jacket, located in the lower part of the boiling chamber, facilitates the heat exchange, while the vapor phase flows through a demister and condenses in the installed condenser. At boiling, the temperature reaches about 35 °C (95 °F), while the residual pressure is about 5.3 kPa. To remove the compression and excess heat, a fan set is activated. The distillate is recovered from the receiver and pumped through the ejector by the installed effluent pump to create a vacuum. The pressure variation generated is adequate for extracting both the concentrated brine and the distillate. The distillate receiver incorporates a coil through which the R134A Freon flows to cool the distillate to around 25 °C (77 °F), which in turn enhances the ejector’s performance. The installed solenoid valve automatically breaks the vacuum upon completion of the duty cycle, while the integrated check valve keeps the vacuum in the unit when it is in standby mode. The concentrated brine is released via the outflow valve, which opens automatically at the end of the duty cycle.

### 2.4. Configurations for Calculations

Table 3 presents the methods used to measure the physicochemical parameters of the samples. Parameters not included in this table were obtained from analyses conducted by the laboratory of the Larnaca WWTP.

#### 2.4.1. Membrane Units

The operational evaluation of the NF and RO systems followed the instructions of the technical manual of Dupont [46]. To calculate the water recovery (*R*), Equation (1) was used, and for the salt rejection or rejection coefficient (*Rs*%), Equation (2) was employed.

(1) $$R\;(\%)=\frac{Qp}{Qf}\times 100$$

(2) $$Rs\%=\left(1-\frac{Cp}{Cf}\right)\times 100$$

where *Qp* and *Qf* are the permeate flow and the feed flow, respectively, in L/h. *Cp* and *Cf* are the concentrations (mg/L) of permeate and feed solutions determined from analyzing the samples.

The permeate flux [47] (f, L/(m^2^ × h)) is calculated with the following Equation (3):

(3) $$f=\frac{Qper}{A\times T}$$

where Qper, *A*, and *T* represent the filtrated solvents volume (L), the filtration area (m^2^), and the filtration time (h), respectively.

For its conversion in International System of Units (S.I.) the following Equation (4) was used:

(4) $$1\;L/(m^{2}\times \;h)=1.7\times 10^{-5}\;m/s$$

To assess the permeability (f′, L/(m^2^ × h × bar)) of the process, the following Equation (5) was used for its calculation:

(5) $$f^{\prime }=\frac{Qp}{A\times T\times TMP}$$

where *Qp* is the permeate flow rate (in liters, L), A is the membrane area (in square meters, m^2^), *T* is the duration time of the process (in hours, h), and TMP is the transmembrane pressure (in bar).

For its conversion in International System of Units (S.I.) the following equation 6 was used:

(6) $$f^{\prime }L/(m^{2}\times h\times bar)=\frac{f(\frac{m}{s})}{TMP\;(Pa)}$$

The measured pressure drop (Δ*P*, kPa) was calculated using the following Equation (7):

(7) $$\Delta P=\frac{Pf}{Pc}$$

#### 2.4.2. Thermal Units

For the evaluation of the MED evaporator unit and crystallizer, the following equation 10 was used for the calculation of the concentration factor (*CF*) and Equation (8) was used for the water recovery evaluation

(8) $$CF=\frac{Cc}{Ci}$$

where the *Cc* and *Ci* are the concentrations (mg/L) of the systems and inflow respectively.

The salt purity was calculated based on the measures of ion concertation from the following Equation:

$$NaCl\;purity\;(\%)=(\frac{\frac{\left[Na\right]}{ArNa}}{\frac{\left[K\right]}{ArK}+\frac{\left[Na\right]}{ArNa}+\frac{\left[Mg\right]}{ArMg}+\frac{\left[Ca\right]}{ArCa}})\times 100$$

where [*Na*], [*K*], [*Mg*], [*Ca*] is the concentration of sodium, potassium, magnesium, and calcium in mg/L, and Ar_n_ is the atomic mass of the ions.

## 3. Results

### 3.1. Results of Each Operated Technology

#### 3.1.1. Nanofiltration Unit

**Figure 3:**
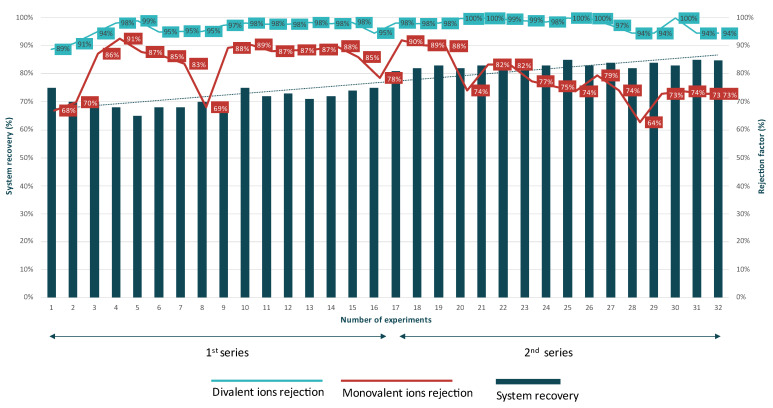
Rejection factors of divalent and monovalent ions in different system recoveries.

The NF system was evaluated through two series of operations that were investigated during the pilot operation. In the first series, the system operated with a recovery rate ranging from 65% to 75%, with an average salinity of the feed wastewater of 3000 mg/L. In the second series, the recovery rate was increased to a range of 75% to 85%, while the feedwater salinity was lower, at around 2500 mg/L. With the divalent and monovalent ion separation being the main focus on the NF unit, as shown in Figure 3, and with the system recovery progressively increasing, the experimental results showcased performance improvements (better separation). The rejection efficiency of divalent ions (Ca^2+^, Mg^2+^, SO_4_^2−^) improved from an average of 96% in the first series to 98% in the second series. Detailed results showed that Ca^2+^, Mg^2+^, and SO_4_^2−^ were rejected at 95%, 93%, and 87%, respectively, in the first series, and the rejection efficiency was improved in the second series with divalent ion rejection ranges reaching 98%, 95%, and 90%.

Regarding the rejection rates of the major monovalent ions K^+^, Na^+^, and Cl^−^, where a lower rejection percentage indicates better separation, the mean rejection decreased from 83% in the first series to 78% in the second series, demonstrating improved selectivity and permeate quality. The rejection rates for K^+^, Na^+^, and Cl^−^ in the first series were 84%, 87%, and 83%, respectively. As system recovery increased, these rejection rates improved to 78%, 84%, and 76%.

Overall, increasing system recovery resulted in better separation of the ions present at the inflow acting as an efficient pre-treatment step of the saline inflow.

**Figure 4:**
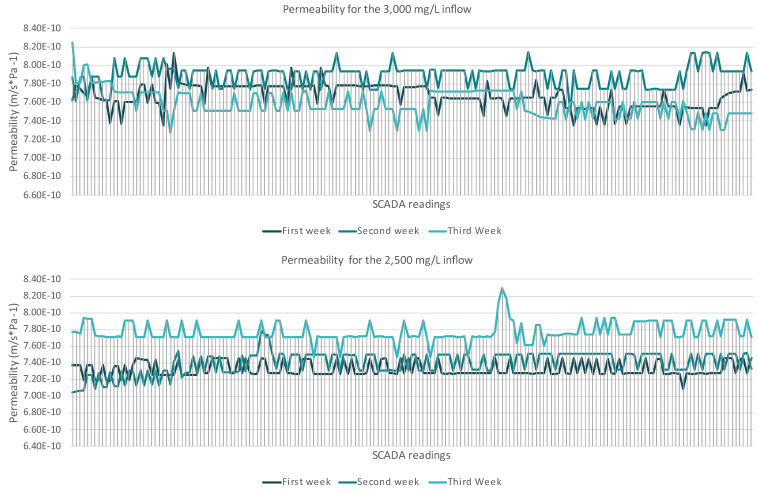
Permeability at different concentrations.

To assess if the high recovery compromised the membrane performance, the permeability and permeate flux of the membrane were measured, since they are the two key parameters that play a crucial role in evaluating membrane performance during the filtration processes. The data presented refer to a three-week stable operation without any reported issues. The discussed system operated for two and a half years at the Larnaca WWTP, but to avoid high number deviations at the reported values of the operation results, a specific period of time was selected and discussed. In the reported experiments, the inflow pressure remained stable with minor operational adjustments taking place. A comparison between permeability and flux at the first series and the second series allowed us to examine whether increased salinity and high recovery rates contributed to performance deterioration. As presented in Figure 4, the first series showed a gradual decline in permeability over time. The mean values of the permeability (ms^−1^Pa^−1^) of each week were 7.67 × 10^−10^ ms^−1^Pa^−1^ (with SD 1.35 × 10^−11^), 7.89 × 10^−10^ ms^−1^Pa^−1^ (with SD 1.20 × 10^−11^), and 7.59 × 10^−10^ ms^−1^Pa^−1^ (with SD 1.47 × 10^−11^). From the first week of operation to the third week, the permeability declined to around 1%. At the end of the third week, a decline in permeability was observed that could be attributed to scaling formation, with the calculated values being the lowest observed (7.2 × 10^−10^ ms^−1^Pa^−1^).

The second series demonstrated relatively stable permeability. The calculated values remained consistent across all three weeks, with only minor fluctuations. The mean values were for the first week 7.32 × 10^−10^ ms^−1^Pa^−1^ (SD 8.57 × 10^−12^), for the second week 7.40 × 10^−10^ ms^−1^Pa^−1^ (SD 1.34 × 10^−12^), and for the third week 7.77 × 10^−10^ ms^−1^Pa^−1^ (SD 1.16 × 10^−12^). From the first week of operation to the third week of operation, the permeability declined around 6%.

**Figure 5:**
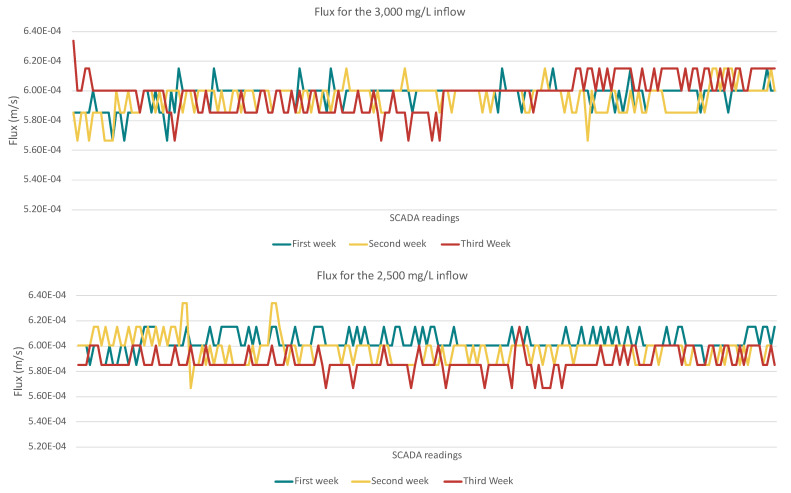
Permeate flux variations at different concentrations.

A similar comparison was also conducted with permeate flux variations under the two different TDS inflow concentrations, revealing key differences in membrane performance over the three-week period (Figure 5). The first scenario showed a high degree of flux fluctuation through the three-week operation, indicating that increased salinity levels introduced higher osmotic resistance, leading to reduced water passage. The mean values of the flux (m/s) through the operation were similar (5.9 × 10^−4^ m/s). The first two weeks, the calculated SDs were 8.6 × 10^−6^ and 9.80 × 10^−6^, and the third week, the SD was 1.18 × 10^−5^. At the final week, the flux ranged from 5.67 × 10^−4^ m/s to 6.34 × 10^−4^ m/s.

In the second scenario, the flux remained relatively stable, fluctuating from 6.67 × 10^−4^ to 6.34 × 10^−4^ m/s with minor variations across all three weeks. During the third week (redline), the lower flux was observed (5.67 × 10^−4^ m/s). The limited flux decline and the low SD, 7.68 × 10^−6^, 1.04 × 10^−6^ and 8.62 × 10^−6^, in this experimental setup indicates that the membrane was performing efficiently, with no immediate signs of significant fouling or scaling.

Following the permeate flux and the permeability, the ΔP was the final membrane performance indicator that as expected followed the trends of permeate flux and permeability. In the first series, the higher ΔP values were recorded at the third week 87 kPa (SD 12), suggesting increased resistance, which, if left unaddressed, could lead to higher energy consumption, reduced water recovery, and long-term membrane degradation. During the second series, the ΔP remained stable at 80 kPa (SD 3). It is worth mentioning that during those 3 weeks, no backflash or chemical cleaning was implemented.

Overall, the second series operated at a higher recovery rate of 75–85% with lower feed salinity, demonstrated better membrane performance. The divalent ion rejection improved, and better ion selectivity was observed. Membrane permeability remained stable over three weeks with mean values of 7.32 × 10^−10^, 7.40 × 10^−10^, and 7.77 × 10^−10^ m·s^−1^·Pa^−1^, and flux ranged from 6.67 × 10^−4^ to 6.34 × 10^−4^ m/s with very low SD values. These results indicate that the second series is more efficient, with reduced membrane fouling risk.

#### 3.1.2. Reverse Osmosis Unit

The NFP flow (softened flow) was treated at the plant by the RO unit with its operation focusing on the rejection factor of the membranes and the optimal operational recovery. The RO unit evaluation was not based on the fluctuation of inflow (as) of NF, but it was focused on the recovery of the higher water quality and quantity since that the main focus of the whole installation. To achieve this, the two-pass operation provided the necessary increase of ion rejection while optimizing water recovery efficiency.

**Figure 6:**
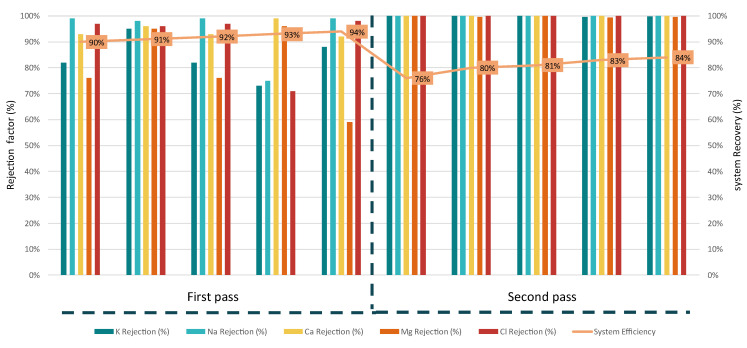
RO operational results for different recoveries.

The average inflow of the RO unit within the experimental circle of RO operation was 690 mg/L with 50% of the overall concentration to be attributed to Cl^−^ ions and 35% to Na^+^ ions. As can be seen for Figure 6, the first-pass RO operation with system recovery ranging from 90% to 94% achieved high rejection rates for Na^+^, Ca^2+^, and Cl^−^, consistently above 90–97%, while Mg and K rejection showed greater variability, with the lowest Mg rejection recorded at 59%. With the second pass, the treated ROC stream was tested with recovery rates ranging at lower levels than the first one since the inflow of the second pass was of higher concentration with an average concentration of 9370 mg/L. At the second pass, the rejection rates improved significantly, with most ions reaching near 99.99% rejection.

The maximum designed permeate flux of the RO system (with the 4 LC-HR 4040), based on the integrated membrane, is 3.65 × 10^−6^ m/s. During the operation at the first stage, the system operated at the maximum permeate flux. The high flux operation significantly impacted membrane performance, fouling tendencies, and ion rejection stability. Operating at 3.22 × 10^−6^ m/s in the first stage, combined with a high recovery rate (90–94%), intensified concentration polarization (CP) near the membrane surface, but since the inflow had low TDS (around 700 mg/L), the risk of scaling or fouling remained low.

The second-pass RO operation that was carried out was operated with lower flux (from 5.10 × 10^−7^ m/s to 5.95 × 10^−7^ m/s), and recovery rates enabled a more stable rejection of ions, with most contaminants reaching near-complete removal (~99.99%). The lower flux in the second pass allowed for better membrane performance, reduced scaling risks, and improved rejection consistency across all ions.

The recovered water from the RO system is of significantly improved quality, with high purity and low dissolved solids, making it suitable for various applications. In the first-pass RO, the permeate achieved a TDS concentration of approximately 35 mg/L, lowering hardness by removing Ca^2+^ and Mg^2+^ while reducing the Na^+^ and Cl^−^ concentrations. The same results were also measured for the second-pass RO with average water quality of 36 mg/L, with rejection rates reaching 99.9% for all the ions. This high-quality recovered water can be used directly in boiler feed systems, pharmaceutical manufacturing, semiconductor production, or as irrigation water after re-mineralization.

From the system operation measurements, it was determined that the first-pass RO required moderate feed pressure (~400–800 kPa for 600 mg/L TDS), resulting in relatively low energy consumption. The energy demand ranged from 0.5 kWh/m^3^ of feed at 90% recovery to 0.8 kWh/m^3^ of feed at 93% recovery, which is significantly lower compared to high-salinity applications. However, in the second-pass RO, the feed TDS increased significantly to around 9000 mg/L, leading to a rise in osmotic pressure and, consequently, higher energy consumption. The energy demand in this stage ranged from 1.6 kWh/m^3^ of feed at 76% recovery to 2.0 kWh/m^3^ of feed at 84% recovery.

To evaluate the potential for scaling during the first and second passes of the reverse osmosis (RO) process, the Langelier Saturation Index (LSI) was calculated for the concentrated streams (RO concentrate) of each pass. Although bicarbonates (HCO_3_^−^) were not directly measured in the concentrate, data provided by the Larnaca Wastewater Treatment Plant (WWTP) indicated that the influent to the unit contains approximately 230 mg/L of HCO_3_^−^. Assuming that the nanofiltration (NF) unit achieves a rejection rate greater than 95% for bicarbonate, the concentration in the RO concentrate streams is expected to be no higher than approximately 2 mg/L. Based on these assumptions, the LSI was calculated and found to be negative in both cases: −2.96 for the first-pass RO concentrate and −2.44 for the second-pass RO concentrate. These values indicate that the water is undersaturated with respect to CaCO_3_ and thus presents no scaling risk from precipitation, but since it is negative, the solutions are considered corrosive.

To ensure the system’s long-term stability, all the equipment’s pipes were constructed from plastic materials to prevent scaling. During the two-and-a-half-year operation period, no scaling was observed on the equipment.

#### 3.1.3. MED Evaporator Unit

The performance of the MED evaporator was assessed based on water recovery rates, with the goal of achieving the highest possible concentration factors (CF). The experiments involved gradually increasing the duration of brine injection while keeping all the other operating parameters (pressures and temperatures of each effect) as stable as possible. The aim of these experiments was to evaluate how increasing the feed time affected both the water recovery rates and the resulting concentration factors in relation to the energy requirements.

**Figure 7:**
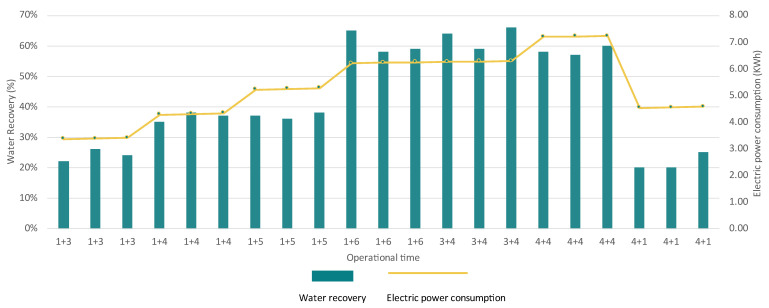
Water recovery efficiency vs. power consumption from the operation of MED.

At the following Figure 7, the brine injection and steady state operation variations are described as follows: Number1 + Number2. The first number indicates the feed time of the system, and the second number indicates the time of steady-state operation. For instance, the notation “1+3” signifies that the brine was fed into the system for 1 h, followed by an operational period of 3 h.

As can be seen from the relationship between water recovery efficiency and electric energy consumption across various operation times, the water recovery increases steadily with operation time combinations such as “1+4”, “1+5”, “1+6”, and “3+4”, peaking at 70% for “1+6” and around 75% for “3+4”. Electric power consumption also gradually increased during operation, particularly with higher water recovery, though the relationship was not perfectly linear. As an example, the “1+6” and “3+4” combinations demonstrated a balance of high-water recovery and moderate energy consumption, indicating greater efficiency. In contrast, the “4+1” operation showcased low water recovery (20–25%) while consuming high levels of electric power, highlighting inefficient operation.

**Figure 8:**
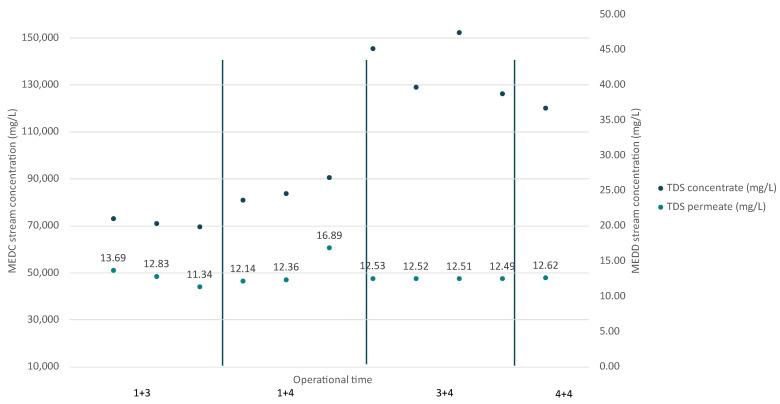
TDS of the MED concentrate stream and distilled water.

The MED evaporator system demonstrated effective separation of dissolved solids, consistently producing high-quality condensate with very low TDS across all operational times (Figure 8). In the 1+3 phase, the inlet feedwater TDS ranged from 52,563 mg/L to 56,947 mg/L, with condensate TDS reduced to 11.34–13.69 mg/L, while the concentrate reached up to 73,009 mg/L, indicating efficient salt concentration. The 1+4 phase showed similar trends, with feedwater TDS ranging from 56,997 mg/L to 51,967 mg/L, and the concentrate reaching 90,471 mg/L. During the 3+4 operation, the feedwater TDS reached up to around 55,000 mg/L, and with the condensate remaining pure (TDS: 12.50 mg/L), the concentrate reached more than 150,000 mg/L. The 4+4 configuration with feedwater TDS levels of around 52,000 mg/L and the condensate maintaining a low TDS value reached a high system recovery of around 60%.

With the two best operations being “3+4” and “4+4”, the “3+4” configuration showcased more stable performance (SD = 2% of the CF and SD = 0.01 of water TDS) compared to “4+4”. For that reason, “3+4” was selected for continued operation at the plant, as it consistently achieved water recoveries of 65% with a distilled water quality of up to 12.55 mg/L. This operational configuration will be named as “MEDbest” for the following analysis.

During the operation of the MEDbest system, various operating parameters were monitored to evaluate its performance. The evaporator was designed to operate under vacuum conditions, ensuring efficient heat transfer and water evaporation at lower temperatures. Throughout the process, the first effect operated at approximately 38 kPa while the second effect functioned at 28 kPa.

The thermal performance analysis demonstrated that the brine was effectively pre-heated as it moved through the system. Initially, the brine inflow temperature at the experimental setup was 27.2 °C, gradually increasing to a peak of 73.6 °C. At the pilot scale, the inflow was pre-heated using a tube-in-tube heat exchanger, where steam from the second effect transferred heat to the incoming brine.

The boiler inlet temperature was also monitored throughout the operation. It fluctuated with 87.6 °C at the start and gradually decreased to 73.0 °C. Meanwhile, the boiler outlet temperature remained consistently lower than the inlet temperature, starting at 40.3 °C and rising to 60.8 °C towards the end. This confirms that the cold brine effectively absorbed heat through contact with hot water, optimizing thermal energy utilization for the inflow heating process.

#### 3.1.4. Vacuum Crystallizer Unit

The crystallizer unit was tested using the concentrate stream from the MED evaporator (MEDC) with the 3+4 configuration, with the goal of achieving the saturation point of sodium chloride (24 to 26%) and recovering solid salt. The operational study assessed the impact of different operational durations (4, 6, 8, and 10 h), and since the system is fully automated, there were not any parameters that could be adjusted during the operation. The performance evaluation of the system was based on the correlation of the operational hours and how those influenced water recovery, salt purity, and energy consumption in batch mode operation.

Among the tested configurations (Table 4), the 10 h operational period achieved the best overall results, achieving the optimal water recovery, and improved energy efficiency. However, residual salt deposition on the internal surfaces of the crystallizer was observed, which could negatively impact long-term operation. This accumulation of salt particles on internal walls was primarily caused by supersaturation of the brine, temperature fluctuations, and solute concentration changes. Therefore, while the 10 h operation provided the best performance, the potential drawbacks related to scaling and increased maintenance demands must be considered for long-term system optimization.

The nine-hour configuration produced the highest purity salt (max 85%) with moderate water recovery, while the eight-hour operation resulted in slightly lower NaCl purity (max 83%), but since the salt purity highlights the efficiency of the other systems (rejection from NF and RO) the eight-hour configuration emerged as the preferred choice for balancing water recovery with moderate energy consumption. Within those 8 hrs of operation, the VC unit achieved 65% water recovery (Table 5).

**Figure 9:**
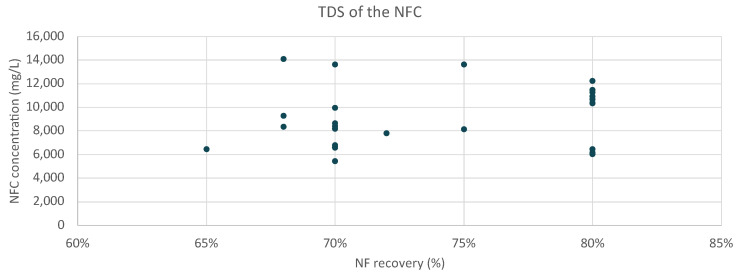
Concentration of the NFC flow treated by the VC.

The NFc was also treated by the installed VC unit at the plant recovering mixed salts containing mainly calcium and magnesium. The following Figure 9 presents the TDS (based on the major ions) that was treated by the VC unit at the plant.

Throughout the operation, the VC unit demonstrated a consistent ability to handle varying salinity levels, but during the operation, the system encountered operational challenges due to the existence of high concentrations of calcium and magnesium. These divalent ions led to the precipitation of salts inside the boiling chamber, forming scale on the heat transfer surfaces. Those formations were caused by the CaSO_4_ and CaCO_3_ that due to lower solubility from the other ions on the inflow were deposited and formed, creating sparingly soluble salt crystals. As a result, the installed unit had to operate less hours, around 5 (achieving 70% recovery), producing a concentrated stream (slurry) that was afterward dried with solar drying. After the drying process, the resulting salt contained 46% Ca salts (with mainly SO_4_, Cl), 13% Mg salts (with mainly SO_4_, Cl), and 38% Na salts.

The recovered sodium chloride (NaCl) in the pilot-scale study did not reach high purity levels. This limitation is primarily due to the nanofiltration (NF) unit, which did not achieve complete rejection of divalent ions during operation. The rejection efficiency is attributed to the specific membrane used in the pilot installation. In a full-scale implementation, this issue could be mitigated by selecting a more appropriate membrane with lower monovalent ion rejection, thereby enabling the recovery of NaCl with greater purity. If a sufficiently high purity is achieved, the recovered salt could potentially be reused in the plant’s chlorination stage during disinfection. Currently, the plant consumes approximately 390 tons of NaCl per year; thus, integrating high-purity salt recovery could significantly reduce operating costs for the plant. At its present purity level, however, the recovered salt could be marketable at a low price, estimated around €20 per ton after discussion with market actors. Regarding the mixed salt (VCNFSalt), to avoid the landfilling and to enhance the circular economy, it could be given free of charge in companies that can utilize it.

## 4. Discussion

### 4.1. Discussion of ZLD System Results

#### 4.1.1. Membrane Units

NF membranes have been studied for their application in brackish water treatment, particularly with regard to permeability, flux, and ion rejection efficiency. According to Du et al. [48], the NF membrane VNF2, operating with a 10 g/L saline feed at a flow rate of 2 m^3^/h and an applied pressure of 15.5 bar, achieved a flux of 41.9 L/(m^2^h). Furthermore, Altaee et al. [49] reported that depending on feed salinity, NF membrane systems can attain recovery rates of approximately 75% or higher. In addition, Hudaib et al. [50] investigated low-pressure ceramic TiO_2_ NF membranes and observed a significant reduction in pure water flux when treating salt mixtures, due to the presence of divalent ions such as calcium and magnesium. The study found a rejection order of Cl^−^ > Ca^2+^ > Na^+^ > Mg^2+^. Similarly, research by Ruiz-García et al. [51] highlighted that NF membranes are well suited for applications requiring tailored permeate compositions—such as irrigation—thanks to their ability to offer both selective ion removal and energy efficiency.

The study by Mohamed E.A. Ali focused on the application of NF membranes to treat brine discharge from RO desalination plants, with the NF membranes achieving 96–98% removal of total hardness and 79–89% of TDS, acting as an effective pretreatment stage of the RO unit [52]. Park et al. [53] also showed that the NF membranes were able to remove the divalent cations completely from the brine from seawater RO.

The integration of NF-RO hybrid systems has been extensively studied for various water treatment applications, demonstrating significant improvements in ion rejection, energy efficiency, and water recovery. A study by Fatima Elazhar [54] showcased the integration of the NF-RO hybrid process, proving its huge potential to be widely applied for hardness removal and compared the energy performance of a conventional NF-NF system with a hybrid NF-RO system for hardness removal. The hybrid NF-RO system, when combined with a blending strategy, was found to be simpler and more energy-efficient and can achieve a high overall water recovery rate of 95%, with improved permeate quality (lower TDS), and an RO stage recovery of up to 80%. A blending portion of 32.45% NF brine with RO permeate (240 L/h) met drinking water quality standards in the conducted analysis. The inclusion of NF upstream increased the overall water recovery rate to 95%, with the RO stage achieving up to 80% recovery when treating less saline feedwater [55].

#### 4.1.2. Thermal Units

The study by Giuseppe Scelfo et al. demonstrated the effective use of a two-effect MED pilot plant, powered entirely by low-grade waste heat, as a brine concentrator within a Zero Liquid Discharge (ZLD) framework. Operating with NF permeate as feedwater, the unit achieved a maximum concentration factor (CF) of 8 and recovery ratios exceeding 85%, even at relatively low top brine temperatures (TBT) between 43–50 °C. The produced distillate consistently showed high quality (conductivity < 25 μS/cm), while the brine was concentrated near saturation, making it suitable for further treatment via crystallization or evaporation ponds [44]. The study by Ortega-Delgado et al. [56] developed a detailed thermodynamic model of forward-feed multi-effect distillation (MED-FF), showing that increasing the top brine temperature (TBT) from 70 °C to 120 °C could significantly enhance the gain output ratio (GOR) by ~70% and reduce the specific thermal energy consumption (SEC) by 45%. Xevgenos et al. [57] reviewed brine management strategies, highlighting MED’s role in hybrid systems (e.g., with RO or crystallizers) for maximizing recovery and reducing environmental discharge, particularly due to MED’s modularity, scalability, and compatibility with thermal integration. Drioli et al. [58] emphasized the importance of MED in hybrid configurations for sustainable brine management and circular water economy strategies, especially when driven by renewable or waste heat sources to concentrate brines for crystallization. Lastly, Atieh et al. [59] underscored MED’s effectiveness in industrial water reuse applications, particularly for high-salinity brines, due to its robustness, compatibility with ZLD goals, and potential for integration with other treatment technologies such as membranes or thermal crystallizers, positioning MED as a core component in advanced brine minimization frameworks.

The salt recovery methods usually employ solar evaporation and/or thermal/mechanical evaporation [60]. Different studies have evaluated the operation of different types of crystallizers aiming at recovering salts from saline flows. The study by Kristofer Poirier [61] showcased the recovery of NaCl of high purity (near 100%) from seawater brines recovering the 99.2% of water of the water content. Robert L. McGinnis [62], from the operation of the thermal crystallizer unit, concentrated the brine up to 180,000 ± 19,000 mg/L TDS, recovering water with 300 ± 115 mg/L TDS. Panagopoulos et al. state that the brine crystallizer can achieve up to 99% water recovery and consume between 52–70 kWh/m^3^ of energy for feed brine with a TDS up to 300,000 mg/L. Additionally, the freshwater produced has a high quality with a TDS lower than 20 mg/L [63]. With the thermal crystallizers being a viable technological solution, its high energy consumption is one that could hinder its wider implementation.

### 4.2. Overall Evaluation of the ZLD System

**Figure 10:**
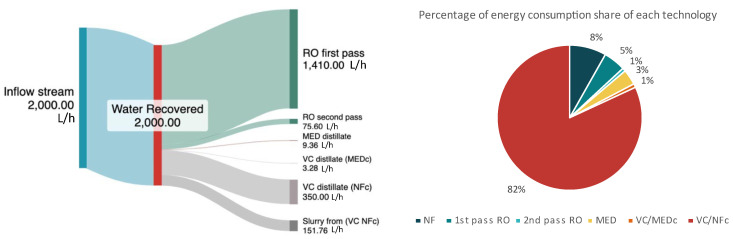
(**Left**) Water recovery of each system, (**Right**) energy consumption of each system for the 2 m^3^/h of ZLD inflow.

The overall energy consumption of the ZLD system was measured at the plant of around 12.20 kWh/m^3^ of the ZLD inflow, with an operational flow rate of 2 m^3^/h. The integrated thermal technologies accounted for approximately 86% of the total electrical energy consumed. As expected, the MED evaporator, while crucial for treating high-salinity streams, required the highest energy input, yet contributing to the low volume of recovered water (as illustrated in Figure 10). In contrast, the RO system, particularly during the first pass, was responsible for recovering the largest quantity of water, accounting for 71% of the total recovered water, while consuming only 1.38 kWh—highlighting its efficiency in handling the inflow with relatively low energy input.

For thermal technologies such as MED, the total energy consumption is typically calculated with the respective thermal (primary) and electrical (secondary) energy inputs, with reported values ranging between 40 and 80 kWh/m^3^ for thermal energy and 2 and 5 kWh/m^3^ for electrical energy consumption [64]. In the case of this pilot unit, the thermal energy required for concentrating the inflow was indirectly measured as electrical energy, since the boiler used to supply heat to the first effects was connected to the plant’s control panel. As a result, the boiler’s energy consumption along with the other parts of the system (pumps, electrical valves) was recorded as electrical input, resulting in an average of 60 kWh/m^3^ of MED unit inlet.

ZLD systems can be integrated based on the employed technologies to (a) thermal ZLD systems (pre-treatment, brine concentrator (BC) brine crystallizer (BCr)), (b) reverse osmosis-incorporated thermal ZLD technique (thermal ZLD system and RO), and (c) emerging membrane-based ZLD systems (with electrodialysis, or/and forward osmosis, or/and membrane distillation) [65]. Based on the inflow characteristics and the achieved results, the energy consumption of a ZLD system can vary from 20 to 200 kWh/m^3^ of recovered product [66,67]. Elginoz et.al conducted a Life Cycle Assessment (LCA) analysis of a ZLD system incorporating NF, RO, ED, Cr, and ion exchange membranes (IEX) consuming 11 kWh/m^3^ of brine inflow [68]. Results from a technoeconomic analysis conducted by Panagopoulos showcased for a ZLD system (incorporating RO, BC, and BCr) an energy consumption of about 500 kWh/m^3^ of inflow and for a ZLD system (incorporating RO, three BC, one BCr, and NF unit), an energy consumption around 700 kWh/m^3^ of inflow [69]. With the salinity usually being the main influential parameter of the energy requirement, the thermal technologies consume significantly more energy than membrane-based processes, ranging from 7–30 kWh/m^3^ of recovered water for BCs and 50–60 kWh/m^3^ of recovered water, for BCrs [70].

For the 2 m^3^/h of inflow form the Larnaca UWWTP, the overall energy consumption was measured around 24.42 kWh, with each system operating at is optimal conditions as shown in Table 6.

#### 4.2.1. Chemical Use During the Operation

For the cleaning of the NF and RO systems, both acid and alkaline clean-in-place (CIP) procedures were implemented. CIP was initiated when the transmembrane pressure (Δp) exceeded 0.90 bar—close to the maximum permittable Δp of 1.0 bar for the NF 90-4040 and LC-HR-4040 membranes—or when a pressure increase of more than 10% was observed during operation. The acid cleaning involved the circulation of a 30% HCl solution at high flow rate and low pressure for 30 min. Similarly, alkaline cleaning was performed by circulating a 50% NaOH solution through the membranes for 30 min. Over the course of the cleaning operations, a total of 0.0022 L (OF HCl)/m^3^ of inflow and 0.0015 L (of NaOH)/m^3^ of inflow were consumed.

Since the pH fluctuated from 8 to 10 during operation, a pH adjustment was implemented to inflow to protect the NF membranes and to prevent scaling and fouling. A mean value of 700 mL/m^3^ of inflow of 30% HCl was measured to adjust the inflow to pH of around 6.

For the cleaning of the two thermal units, the distillate water produced by the MED evaporator was used as the cleaning medium for both systems.

#### 4.2.2. Recovered Water from the Pilot Unit

The primary intended use for the reclaimed water in this case study is agricultural irrigation, which aligns with its predominant use across Cyprus, aside from limited municipal applications. As the reclaimed water from the Larnaca WWTP is already utilized for irrigation purposes, microbiological quality parameters are considered non-critical and compliant with existing standards.

The main challenge in the reclaimed water was the high salinity, which poses potential risks to soil and crop health. In addition, phosphorus content could present another area of concern due to its potential to cause membrane fouling in desalination or polishing processes.

The use of recycled water for irrigation from wastewater treatment plants is regulated at the EU level by the Urban Wastewater Treatment Directive (91/271/EEC), which aims to ensure the proper collection, treatment, and reuse of urban wastewater in order to protect public health and water bodies. Furthermore, the water quality characteristics are set by the Regulation (EU) 2020/741, which sets minimum requirements for water reuse, focusing on water quality monitoring and risk management for safe agricultural irrigation. In Cyprus, the main legislation that has been implemented to adapt to the EU policy framework is the Code of Good Agricultural Practice, which regulates the use of recycled water for irrigation and specifies the types of crops that can be irrigated with the reclaimed water. Since tertiary treatment is mandatory in Cyprus regardless of its use (irrigation, aquifer recharge, discharge to the sea), the Code of Good Agricultural Practice includes a restriction on the type of crops that can be irrigated (irrigation of all crops is allowed except for leafy vegetables, bulbs, and condyles that are eaten raw (e.g., lettuce, carrots, celery, parsley), prioritizing its use on forest trees, fodder crops, orchards, and green areas that are not as profitable for farmers.

The limits for the parameters that need to be measured to assess the quality of the water in Cyprus are based on the limits provided by the Regulation (EU) 2020/741, but since Cyprus is using high quantities of the reclaimed water, which it has set internally (within WDD and UWWTP), there are more strict limits for the use of water that go beyond the EU regulation (Table 7).

The mixed recovered water (Table 8) from the pilot demonstrated high quality and complies with the Cypriot irrigation standards for treated wastewater from UWWTPs serving populations ≥ 2000 p.e. All key parameters—including TDS at 29.3 mg/L and Cl^−^ at 10.77 mg/L—were significantly below the respective regulatory limits (e.g., 300 mg/L for Cl^−^). Nutrients such as TP and TN were not detectable (n.d.), indicating excellent removal performance, attributed to the NF unit. Similar studies have highlighted the high removal efficiency of TN and TP using NF membranes [39,40]. However, these benefits often come with operational trade-offs. In this pilot-scale operation, the potential need for more frequent membrane cleaning cycles and the risk of earlier membrane replacement were not assessed, but these aspects should be considered in full-scale applications. Finally, BOD_5_ was measured at <1 mg/L and SS at 0.2 mg/L—both substantially below the threshold of 10 mg/L.

Based on European standards, *E. coli* (number/100 mL), BOD_5_ (mg/L), TSS (mg/L), and turbidity (NTU) should be measured. *E. coli* and other organic micropollutants or pathogens were not measured in the pilot system, since all possible pathogens are expected to remain in the concentrated stream in the RO system and in thermal processes. In the full-scale implementation of the ZLD system, the recovered water will undergo final treatment at the plant in the form of chlorination, as required by Cypriot law for wastewater treatment plants. This disinfection stage is expected to eliminate any remaining microbial contaminants, thereby ensuring compliance with the required water quality standards for reuse.

Using desalinated water for irrigation directly may lead to deficiencies and plant growth problems, as essential nutrients are removed during these processes. Important quality parameters to consider for desalinated water are the sodium adsorption ratio (SAR), essential nutrients such as calcium, magnesium, and sulfate, calcium to magnesium ratio, chloride and sodium phytotoxicity, and boron phytotoxicity. For that reason, in full scale implementation the re-mineralization could be studied to meet the specific needs of different types of crops.

## 5. Conclusions

The integrated ZLD system demonstrated high operational efficiency and effectiveness in treating the reclaimed saline streams from the UWWTP of Larnaca, with each unit contributing to overall water recovery, brine minimization, and high-quality water production. From the conducted analysis, the second series of NF operation, achieving the higher recovery rate of 75–85% with lower feed salinity, demonstrated better membrane performance. The divalent ion rejection and the ion selectivity were improved. The membrane permeability remained stable over the three weeks with mean values of 7.32 × 10^−10^ ms^−1^·Pa^−1^, 7.40 × 10^−10^ ms^−1^·Pa^−1^, and 7.77 × 10^−10^ ms^−1^·Pa^−1^, and flux ranged from 6.67 × 10^−4^ to 6.34 × 10^−4^ m/s with very low SD values. The NF membrane performance was slightly impacted at higher salinities (3000 mg/L TDS), based on the flux and permeability measurements.

The RO system, particularly the two-pass configuration, achieved recovery rates of 90–94% in the first pass and 76–84% in the second with high ion rejection (round 99.99%) for the major ions including Cl^−^, Na^+^, Ca^2+^, and Mg^2+^ and reduced TDS in the permeate to around 35 mg/L. The system maintained a stable flux performance through its first pass operation (3.22 × 10^−6^ m/s), with the second pass (due to its higher inflow salinity) compromising the permeate flux (5.10 × 10^−7^). The MED unit operated under vacuum conditions, achieving water recovery rates above 65%, under the optimized “3+4” operation. Distillate quality was consistently excellent, with TDS values as low as 12 mg/L. The MED unit, although energy-intensive (~60 kWh/m^3^), effectively handled high-salinity brines and produced stable, high-quality distillate water. Finally, the VC treated the MEDC stream, achieving NaCl purities up to 81% and further concentrating the brine to near-saturation. The best performance was observed during 8 h operations, which achieved stable operation and high-quality water. When treating calcium- and magnesium-rich streams (NFC), scaling became a challenge to the VC unit due to the formation of soluble salts (e.g., CaSO_4_, CaCO_3_), requiring operational adjustments.

The overall energy consumption of the ZLD system was around 12 kWh/m^3^ of inflow, with thermal technologies accounting for 86% of the total energy requirement of the operated ZLD system. The RO system was the most energy-efficient, recovering ~71% of the total water with low energy input (6% of the overall requirements), while the MED and VC units contributed to high salinity reduction and salt recovery but required higher specific energy

Future implementation of the proposed solution should focus on energy efficiency and reducing operating costs. The most energy-intensive components of the system—the MED unit and the VC—could be replaced by a low-temperature evaporator (LTE) that operates more efficiently, reducing energy consumption but also escalating the environmental sustainability of the overall process. In the case of LTE utilization, for every 2 m^3^ ZLD inflow, LTE will treat 0.514 m^3^ of inflow, with an energy consumption varying from 2 to 4 kwh/m^3^ of LTE inlet. So, the energy consumption for the treatments of 1 m^3^ ZLD inflow would vary from 3.89 kWh/m^3^ to 4.41 kWh/m^3^ of ZLD inflow, which is three times lower than the 12.4 kWh/m^3^ of ZLD inflow of the pilot unit. An LTE unit, being a new solution, has been operated up to now in two cases, one being within the framework of the water mining project (project under grant agreement No. 869474), with its results being confidential and available upon request, and the brine mining project (LIFE18 ENV/GR/000019). For this project, it operated as the final crystallization step of a ZLD system that treated saline effluent derived from the coal mining sector [71]. The system, operating under a high vacuum, does not require an external heat source during its operation—only a slight heat input is needed at the start. During operation, it recovers heat, and due to the very high vacuum conditions (up to −980 mbar), if the inflow temperature is around 25 °C, no pre-heating is necessary. The system is fully insulated, with the only energy consumption coming from the electrical power used by the vacuum pump.

In addition, to optimize the final water composition for agricultural reuse, the mixing of the permeate streams with the influent stream could be used to re-mineralize the water, focusing on SAR optimization for specific more profitable crops. From an economic and environmental feasibility perspective, the pilot system demonstrated a promising model, but careful attention must be given to the capital and operational expenditures for full-scale implementation of the system.

## Figures and Tables

**Table 1:** Characteristics of the WWTP effluent (inflow of the ZLD system).

Element	Symbol	Unit	Value (Average/AVE)	Standard Deviation (SD)
Sodium	Na	mg/L	490	20
Potassium	K	mg/L	43	2
Magnesium	Mg	mg/L	61	4
Calcium	Ca	mg/L	149	12
Chloride	Cl	mg/L	890	36
Sulphates	SO4	mg/L	500	40
Tot. Phosphorous	P	mg/L	1.52	0
Tot. Nitrogen	N	mg/L	10.4	0
Elec. conductivity	EC	mS/cm	4.2	1.2
pH	pH		7.9	1
Biochemical Oxygen Demand	BOD_5_	mg/L	<1	0
Chemical Oxygen Demand	COD	mg/L	20	12
Tot. Susp. Solids	TSS	mg/L	7	9

**Table 2:** Properties of the NF and RO membranes [37,38].

	NF Membranes/NF 90-4040	RO Membranes/LC HR 4040
Materials	Polyamide thin-film composite	Polyamide thin-film composite
Permeate flow rate/membrane	7.6 m^3^/d	11 m^3^/day
pH range	2–11	1–13
Max operating pressure	41 bar	41 bar
Salt rejection/membrane	>90%	>99.7%

**Table 3:** Measured physicochemical parameters of the samples.

Physicochemical Parameters	Apparatus	Method
EX SITU		
Na^+^, K^+^, Mg^2+^, Ca^2+^, Cl^−^	Ion chromatograph, Thermo Dionex ICS-5000	ASTM D6919-17
SO_4_^2−^	Ion chromatograph, Thermo Dionex ICS-5000	APHA-AWWA-WEF Method 4110
TDS	Calculation (sum of ionic concentrations)	-
IN SITU		
pH	Elmetron EPX-1t pH electrode or equivalent; Elmetron EPX-4UB or equivalent; Elmetron CX-401 or equivalent	EPA Method 150.1
BOD	BOD bottles (300 mL), incubator, DO meter	Standard Methods for the Examination of Water and Wastewater, 5210 B
Conductivity (mS/cm)	Elmetron ECF-1t; flow sensor	SESDPROC-106-R6

**Table 4:** Results of the recovered products from the VC unit.

Hours of Operation	TDS (mg/L) of Distillate Water	Electric Power Consumption (KWh)
8	12.1	3.84
	10.9	3.46
	12.2	3.72
9	11.1	5.07
	10.8	4.71
	10.2	2.52
	12.5	4.26
	3.6	4.22
10	12.9	5.12
	10.1	4.67
	12.4	4.66
	10.1	5.43

**Table 5:** Salt recovery section concentrated flows.

		MED Unit		Crystallizer Unit	
Element	Symbol	RO Concentrate (After Second Pass) (AVE)	MED Concentrate (AVE)	VC/MEDC (AVE)	VC/NFC (AVE)
		Value	Value	Value	Value
Potassium	K^+^	1822	5101	14,282	605
Sodium	Na^+^	12,381	34,276	94,893	5970
Calcium	Ca^2+^	1239	3517	9987	6259
Magnesium	Mg^2+^	158	437	1209	1056
Chloride	Cl^−^	38,233	107,854	304,257	9913
Sulphates	SO_4_^2−^	59	157	145	4542
pH	pH	6.8	6.6	7.2	7.1
Concentration	TDS	53,897	151,349	424,779	28,351
Volume	m^3^/h	0.0144	0.00504	0.001764	0.15
NaCl purity	%			81	38
System Water Recovery	%	65		65	70

**Table 6:** Overall ZLD energy requirements.

Unit	Operational Parameters	Inlet Unit Volumes (m^3^/h)	Unit Energy Consumption (kWh/m^3^ Unit Inlet)	Energy Consumption per Unit of 2 m^3^ ZLD Inflow (kWh)
NF	Optimal recovery rate: 75% Rejection factors: >99.5% for divalent ions, (84.8–88.4)% for monovalent ions	2	1	2
1st pass RO	Optimal recovery rate: first pass 94% and second pass 84% Rejection Factors: monovalent and divalent ion rejection factor >94.4% at the first and >99.9% at the second pass	1.5	0.8	1.2
2nd pass RO		0.09	2	0.18
MED	Optimal recovery Rate: 65% Operational configuration 3+4	0.014	60	0.84
VC/MEDC	Operational hours: 6 Optimal recovery rate: 63% Purity of salt: NaCl purity 81%	0.005	40	0.2
VC/NFC	Recovery rate: 70% Purity of mixed salt: NaCl purity 38%, consist mainly magnesium and calcium	0.5	40	20

**Table 7:** Irrigation limits of reclaimed water in Cyprus.

Parameters	Limits from Regulation (EU) 2020/741 (Class A)	UWWTPs ≥ 2000 p.e (Applied in Cyprus)	UWWTPs ≤ 2000 p.e. (Applied in Cyprus)				
			All Crops and Green Areas (a)	Vegetables Eaten Cooked (b)	Products for Human Consumption and Green Areas with Limited Access to the Public	Crops for Animal Feed	Industrial Plants
**BOD_5_ mg/L**	10	10	10	10	25	25	25
**COD mg/L**		70	70	70	125	125	125
**Suspended Solids mg/L**	10	10	10	10	35	35	35
***E. coli*** **/100 mL**	5	-	5	50	200	200	200
**Tot. Nitrogen mg/L**	-	15	-	-	-	-	-
**Tot. Phosphorus mg/L**	-	10	-	-	-	-	-
**FOG mg/L**	-	-	5	5	5	5	5
**pH**	-	6.5–8.5	6.5–8.5	6.5–8.5	6.5–8.5	6.5–8.5	6.5–8.5
**Conductivity** **μS/cm**	-	2500	2500	2500	2500	2500	2500
**Cl (mg/L)**	-	300	300	300	300	300	300
**B (mg/L)**	-	-	1	1	1	1	1
**Residual Chlorine** **mg/L**	-	-	2	2	2	2	2

(a) Not for leafy vegetables, bulbs eaten raw, and strawberries. (b) Potatoes, beetroots, etc.

**Table 8:** Analysis of the recovered water at each stage.

	RO 1st Pass	RO 2nd Pass	MED Evaporator	VC/MEDc	VC/NFc	Mixed Recovered Water	UWWTPs ≥ 2000 p.e (Limits Applied in Cyprus)
Ion	mg/L	mg/L	mg/L	mg/L	mg/L	mg/L	
K^+^	2.1	2.1	0.75	0.38	0.45	1.80	
Na^+^	18.3	18.8	1.63	0.92	0.98	14.95	
Ca^2+^	0.7	0.6	1.20	1.48	1.20	0.79	
Mg^2+^	0.3	0.4	0.13	0.89	0.79	0.42	
Cl^−^	12.7	12.8	8	2.94	2.87	10.77	300
SO_4_^2−^	0.7	0.6	0.85	0.38	0.26	0.59	
Tot. Phosphorous	n.d.	n.d.	n.d.	n.d.	n.d.	n.d.	15
Tot. Nitrogen	n.d.	n.d.	n.d.	n.d.	n.d.	n.d.	10
TDS	34.8	35.4	12.6	7.02	6.6	29.3	
BOD_5_	<1	<1	<1	<1	<1	<1	10
COD	n.d	n.d	n.d	n.d	n.d	n.d	70
pH	5.9	6.1	5.7	6.3	6.3	6.7	6.5–8.5
Suspended Solids mg/L	0.5	0.1	n.d	n.d	n.d	0.2	10
Conductivity μS/cm	35 < EC < 50	35 < EC < 50	7 < EC < 10	10 < EC < 20	11 < EC < 18	47	2500

## Data Availability

The authors affirm that the data supporting the findings of this study are accessible and provided within the article.
